# Stimulants

**Published:** 1873-07

**Authors:** 


### STIMULANTS.

A MULE falls down exhausted under an excessive burden; he remains still for some time. You apply the lash and he jumps up in a moment. That lash imparted no additional strength to rise, it only caused the animal to summon to his aid some of the reserve force of the system.

Whiskey, tobacco, tea, coffee, and other so called stimulants, no more add to the strength of the system than did the lash to the exhausted brute. Stimulants no more impart strength to the body than does a check on your bank deposit increase your balance. Bodily strength comes from the food eaten, and can come from no other source. A man may sit down so completely exhausted that he cannot take another step, but let him rest awhile and he steps along quite briskly, because he is stronger—that additional strength having been created while he was resting, by the blood carrying to every point of the body particles of nutriment, each particle imparting its proportion of strength, the aggregate being the power to rise and walk.

Thus it is that the man who drinks liquor frequently during the day cannot, other things being equal, do as much work as his fellow laborer who does not take a drop. All travellers in cold countries know that the more liquor a man drinks to keep him warm the sooner will he freeze.

Liquor makes a man lively, it promotes exhilaration, for it excites the brain and the circulation, but it does this by using the bodily strength in advance, before it was properly prepared for use; that is, the strength of ten o'clock was used at nine, and when ten comes its supply has already been exhausted. Thus, after a drunken spree, a man is so weak that he can scarcely turn himself in bed, and it requires him to rest and sleep for several days, sometimes, before he recovers his natural condition.

So far, then, from the strength or vital force being increased by the use of spirits, tobacco or opium, it is diminished—used up in advance. Hence it is that the best thing in the world a man can do when he is very tired is to rest, and in a little while eat some nourishing food, which will add to the stock of strength on hand.

And yet stimulants are valuable remedies in the hands of the skilful physician, in two ways: they save time; they keep the system up until other means can be brought to bear, as they act with greater rapidity. If a man has been called to extraordinary effort and feels distressingly weak, as, for example, in putting out a fire, or saving another from drowning, he could recover his strength by resting, but if he took some stimulant it would prevent the system from sinking to a very low point—from going as low down as it would otherwise have done, and thus would it require less time to recuperate. But, in all such cases, a cup of hot drink—of ordinary tea—will answer a better and safer purpose than a drink of liquor; and persons sitting down to a meal in an exhausted condition should first of all drink a cup of hot tea and wait a few minutes before eating anything.

